# Noise-resistant developmental reproducibility in vertebrate somite formation

**DOI:** 10.1371/journal.pcbi.1006579

**Published:** 2019-02-04

**Authors:** Honda Naoki, Ryutaro Akiyama, Dini Wahyu Kartika Sari, Shin Ishii, Yasumasa Bessho, Takaaki Matsui

**Affiliations:** 1 Laboratory of Theoretical Biology, Research Center for Dynamic Living Systems, Graduate School of Biostudies, Kyoto University, Yoshidakonoecho, Sakyo, Kyoto, Japan; 2 Gene Regulation Research, Division of Biological Science, Nara Institute of Science and Technology, Takayama, Nara, Japan; 3 Graduate School of Informatics, Kyoto University, Yoshidahonmachi, Sakyo, Kyoto, Japan; Northeastern University, UNITED STATES

## Abstract

The reproducibility of embryonic development is remarkable, although molecular processes are intrinsically stochastic at the single-cell level. How the multicellular system resists the inevitable noise to acquire developmental reproducibility constitutes a fundamental question in developmental biology. Toward this end, we focused on vertebrate somitogenesis as a representative system, because somites are repeatedly reproduced within a single embryo whereas such reproducibility is lost in segmentation clock gene-deficient embryos. However, the effect of noise on developmental reproducibility has not been fully investigated, because of the technical difficulty in manipulating the noise intensity in experiments. In this study, we developed a computational model of ERK-mediated somitogenesis, in which bistable ERK activity is regulated by an FGF gradient, cell-cell communication, and the segmentation clock, subject to the intrinsic noise. The model simulation generated our previous *in vivo* observation that the ERK activity was distributed in a step-like gradient in the presomitic mesoderm, and its boundary was posteriorly shifted by the clock in a stepwise manner, leading to regular somite formation. Here, we showed that this somite regularity was robustly maintained against the noise. Removing the clock from the model predicted that the stepwise shift of the ERK activity occurs at irregular timing with irregular distance owing to the noise, resulting in somite size variation. This model prediction was recently confirmed by live imaging of ERK activity in zebrafish embryos. Through theoretical analysis, we presented a mechanism by which the clock reduces the inherent somite irregularity observed in clock-deficient embryos. Therefore, this study indicates a novel role of the segmentation clock in noise-resistant developmental reproducibility.

## Author summary

The segmentation clock has been widely considered vital for somite formation, because clock-deficient embryos display severe segmental defects. However, irregular somites are still formed, suggesting that the clock is not required for somite formation itself but rather endows it with developmental reproducibility. Thus, the following questions arose: How do irregular somites emerge in a clock-independent manner? How is the irregularity reduced in the presence of the clock? To address these questions, we developed a computational model of somitogenesis. We then clarified that the intrinsic noise induces spontaneous formation of irregular-sized somites in the absence of the clock, and that the clock plays an important role in suppressing the noise effect for reproducible somite formation.

## Introduction

**Fig 1:**
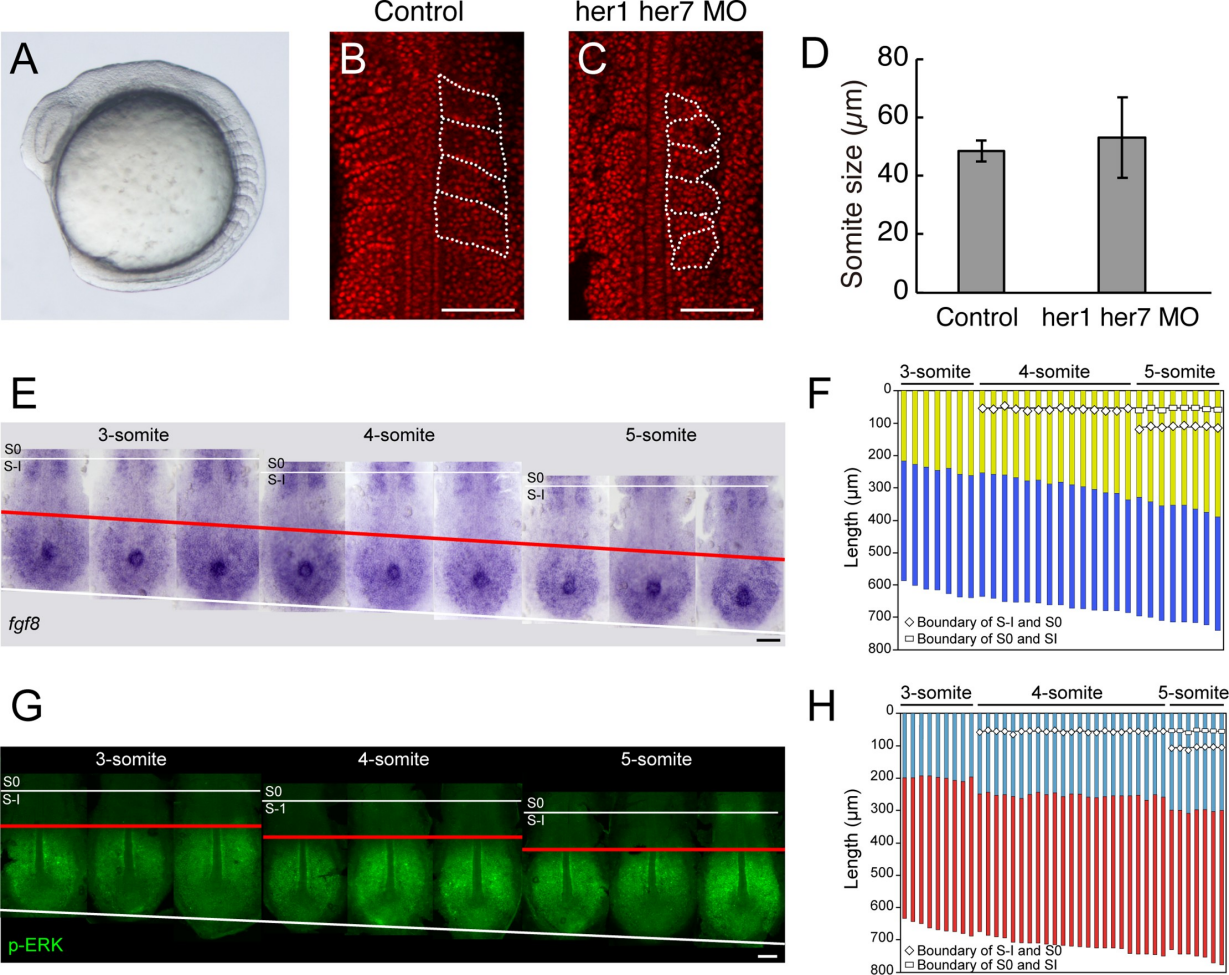
Somite reproducibility and stepwise ERK activity shift for somite formation. **(A)** Lateral view of a wild-type zebrafish embryo during somitogenesis. **(B, C)** Somite morphology in wild-type (B) or clock-deficient embryos co-injected with *her1* and *her7* morpholinos. Nuclei are stained by propidium iodide (red). Somites are outlined by dotted lines. Scale bar: 100 μm. **(D)** Somite sizes in control and clock-deficient embryos (n = 16 each). The somite size variation in clock-deficient embryos (61.1 ± 16.9 μm; C.V. = 0.28) was larger than that in control embryos (50.3 ± 3.6 μm; C.V. = 0.07). Data represent the means and standard deviations. **(E)** Representative dorsal view of PSM *fgf8a* mRNA expression at 3- to 5-somite stages. Red line, *fgf8a* gradient boundary. Scale bars: 100 μm. **(F)** Quantitative presentation of *fgf8a* expression. Embryos (n = 29) were arranged in order of developmental stages, as estimated by somite number and PSM length. High (purple stripe) and low (yellow stripe) *fgf8a* expression domains in each embryo. **(G)** Representative dorsal view of PSM pERK distribution at 3- to 5-somite stages. Red line, pERK boundary. **(H)** Quantitative presentation of pERK distribution. Embryos (n = 39) were arranged as in (*F*). ON (red stripe) and OFF (blue stripe) ERK activity regions in each embryo. Panels *B*, *C*, and *E-H* are adapted with permission from our previous paper [5].

Embryonic development in multicellular organisms is highly reproducible. Conversely, biochemical reactions at the single-cell level are intrinsically stochastic owing to the low copy numbers of expressed molecules [1–3]. Thus, it is believed that organisms have a homeostatic mechanism that can resist the stochasticity of cells, while maintaining the precision and reproducibility required for normal development [4]. However, how the noise affects development *in vivo* remains unclear, because of the technical difficulty in manipulating the noise intensity in experiments. We approached this issue through computational modeling, especially focusing on vertebrate somitogenesis as a model biological system. Equal-sized somites are continuously produced within a single embryo (**Fig 1A and 1B**); thus, reproducibility can be easily evaluated in vertebrate somitogenesis within single, rather than among different, embryos.

Somites are generated by periodic segmentation of the anterior end of the presomitic mesoderm (PSM), which serves as the foundation for the metameric structures of the vertebrate body [6–8]. The most important concept of somitogenesis is the clock and wavefront (CW) model proposed in the 1970s [9]. This model comprises two signals; namely, the oscillatory clock and posteriorly traveling wavefront in the growing PSM. It predicts that, when the wavefront meets the clock signal, PSM cells start to differentiate into somite cells, leading to somite individualization. After extensive investigation, the molecular bases of CW were identified in various vertebrates [7,10]. Clock genes show oscillatory expression in the PSM: *Hes7* (a *Hes* family transcription factor) and *Lunatic fringe* (*Lfng*, a Notch effector) in the mouse [11,12], and *her1* and *her7* (*Hes* family transcription factors) and *deltaC* (a Notch ligand) in the zebrafish [13,14]. Furthermore, morphogen gradients in the PSM function as the wavefront; fibroblast growth factor (FGF) and Wnt signaling produce gradients that weaken at the anterior PSM and continuously move toward the posterior end alongside tail elongation [7,15–18].

However, the effect of innate noise on somitogenesis developmental reproducibility has been outside of the scope of the CW model. Thus, how anti-noise robustness can be acquired remains as an important question for both improving the CW model and elucidating a strategy of noise-resistant developmental reproducibility. Loss-of-function phenotypes of clock genes may provide clues for this issue; clock-deficient mouse (*Hes7*-deficient) and zebrafish (*her1* and *her7* double morphants and/or mutants) embryos still form somites, albeit with variable size (**1C and 1D**), resulting in severe skeletal and segmental defects [5,11,19]. This suggests that the clock is not essential for somite formation itself, but somehow controls somite size regularity by reducing the noise effect, and that noise may induce variably-sized somite formation in a clock-independent manner. Currently, it remains unclear how irregular somites form in clock-deficient embryos and how their irregularity is suppressed in control embryos.

To understand how segmental pre-patterns are determined, we previously investigated FGF/ERK signaling by collecting stained zebrafish embryos [5]. We found that the gentle *fgf8a* gradient (wavefront) was converted into a step-like gradient of ERK activity with a sharp border, which was regularly shifted by the segmentation clock in a stepwise manner (**Fig 1E–1H**). This suggested that ERK integrates clock and wavefront signals and establishes a stepwise pattern within the uniform PSM, which is required for somite individualization. However, an ERK activity stepwise pattern could not be detected in clock-deficient embryos (*her1* and *her7* double morphants), as the spatiotemporal resolution was quite low in our static analyses.

The aim of this study was to clarify how developmental reproducibility is acquired against inevitable noise in somitogenesis. By developing a computational model of ERK-mediated somitogenesis, we investigated the effect of the noise during somitogenesis. This model generated the stepwise ERK activity shift as observed in the PSM [5], which was robust against the noise. It also predicted that the shift occurred even in the absence of the clock, which could not be observed in our previous static analyses, although the timing and distance were irregular owing to the noise, resulting in reduced somite reproducibility. In summary, this study uncovered the clock-independent mechanism of irregular somite formation and also proposed a novel concept for a clock-dependent mechanism of noise-resistant developmental reproducibility in somite formation.

## Models

### Modeling of ERK activity

**Fig 2:**
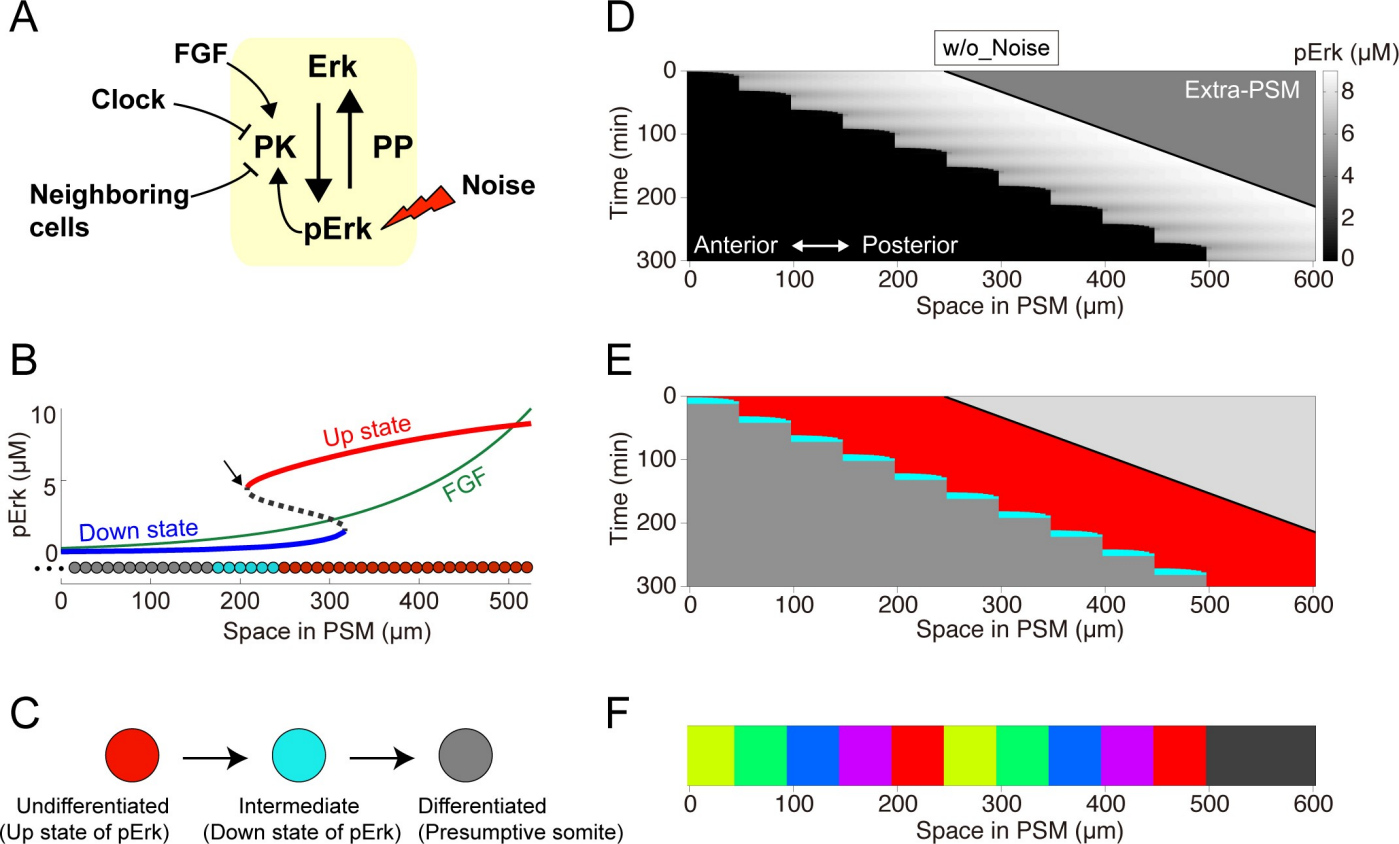
Model of ERK-mediated somite formation. **(A)** Schematic representation of ERK signaling. Our model includes the FGF gradient, positive feedback, segmentation clock, and cell-cell interaction. **(B)** Spatial bifurcation diagram of FGF-dependent ERK activity in the PSM. Up (red line) and down (blue line) ERK activity states. Black dashed line, unstable point between the up and down states; green line, FGF gradient. Because newly formed PSM cells are exposed to high FGF concentrations, ERK activity is in the up state. When cells reach the bifurcation point (arrow), ERK activity rapidly shifts to the down state. **(C)** Cells are initially undifferentiated when ERK activity is in the up state. ERK activity decreases upon up-down transition, promoting cell entry into the intermediate state. The intermediate cell cohort collectively differentiates into presumptive somites if the up-down ERK activity state transition does not occur within a specific time interval. **(D-F)** Simulation results of ERK activity (D), state transitions (E), and the resultant somites (F). (E) Red, cyan, and gray colors indicate undifferentiated, intermediate, and differentiated states, respectively.

We first developed a computational model of ERK-mediated somitogenesis based on previous experimental findings (**Fig 2A–2C**). The model described the ERK activity in one-dimensionally arrayed PSM cells. Depending on tail elongation, newly proliferated PSM cells are added to the posterior end so that their relative positions gradually move toward anteriorly (**Fig 2B**) [7]. ERK is regulated by several factors—the FGF gradient, positive feedback, cell-cell interactions, and the clock—and perturbed by reaction noise (**Fig 2A**).

ERK is activated and inhibited by protein kinase (PK) and protein phosphatase (PP), respectively. Dynamics of phosphorylated ERK in each cell are described by

$$\frac{d[pErk_{i}]}{dt}=\frac{kcat_{PK}PK_{i}(•)[Erk_{i}]}{Km_{PK}+[Erk_{i}]}-\frac{kcat_{PP}PP[pErk_{i}]}{Km_{PP}+[pErk_{i}]}+\sqrt{w}\xi_{i},$$ (1)

where *pErk*_*i*_ indicates the ERK activity level of cell *i*; *kcat*_*l*_ and *Km*_*l*_ (*l*∈{*PK*, *PP*}) are catalytic reaction rates and Michaelis-Menten constants, respectively; *PK*_*i*_(•) is the active protein kinase concentration described by Eq (2); *PP* is the active protein phosphatase concentration; and *ξ*_*i*_ and *w* indicate the white noise (<*ξ*_*i*_(*t*)*ξ*_*i*_(*t’*)> = *δ*(*t*−*t’*)) and noise variance, respectively. The total ERK amount is assumed to be constant as *Erk*_*tot*_ = [*Erk*_*i*_] + [*pErk*_*i*_].

PK is regulated by the FGF gradient, the segmentation clock, positive feedback from phosphorylated ERK, and cell-cell interactions. Regulation was assumed at quasi-equilibrium, so that the PK activity is instantaneously determined by

$$PK_{i}\left(\left[pErk_{i}],[FGF](x_{i},t),[Clock](x_{i},t),\{S_{j}\right\}\right)=a\frac{{\left[pErk_{i}\right]}^{h}}{K^{h}+{\left[pErk_{i}\right]}^{h}}+b[FGF](x_{i},t)-c[Clock](t)-d\sum_{j\in \chi_{i}}S_{j},$$ (2)

where *x*_*i*_ and *t* indicate the position of cell *i* and time, respectively, and *a*, *b*, *c*, and *d* indicate positive constants. The first term represents the positive feedback loop, in which ERK activity further up-regulates PK. *K* and *h* indicate the [*pErk*] concentration required for the half-maximal activation of positive feedback and the Hill coefficient, respectively. The second term represents the FGF signal. The FGF gradient is formed through the production of FGF at the posterior end and degradation over time and continuously moves toward the posterior end alongside tail elongation [20] (**Fig 1E and 1F**). Thus, the FGF signal at position *x* (the origin is fixed at a specific anterior point) is represented by

$$[FGF](x,t)=FGF_{tail}\exp\left[-k_{d}\frac{L_{PSM}(t)-x}{v}\right],$$ (3)

where *FGF*_*tail*_, *k*_*d*_, and *v* denote the tail peak concentration, FGF degradation rate, and tail elongation speed, respectively. PSM growth is represented by *L*_*PSM*_(*t*) = *L*_*o*_ + *vt*, where *L*_*o*_ is the initial PSM length. The equation (*L*_*PSM*_(*t*)–*x*)/*v* represents the time spent by the cell at position *x* from birth at the posterior PSM end. The third term represents the effect of periodic ERK activity inhibition through the segmentation clock. The clock signal was assumed to be spatially uniform over the PSM by

$$[Clock](t)=\frac{1}{2}\left\{\sin(2\pi t/T)+1\right\},$$ (4)

where *T* is the oscillation period. The forth term represents cell-cell interaction, which will be described later. Because a cohort of PSM cells collectively can differentiate into somites in a clock-independent manner via cell-cell interactions [21], neighboring PSM cells were assumed to interact with each other in a state-dependent manner (**Fig 2A**). More detail will be mentioned later.

### ERK bistable dynamics in the model

We also assumed that ERK signaling positive feedback generates bistability, with the activity level showing either “up” (activated) or “down” (inactivated) states, following our previous observation that ERK activity exhibited an all-or-none, step-like distribution in the PSM (**Fig 1G and 1H**). We can thus draw a spatial bifurcation diagram of FGF-dependent ERK activity in the PSM (**Fig 2B**) by assuming rapid ERK dynamics, such that the ERK activity quickly converges to near a quasi-equilibrium point. ERK activity is in the up and down states at high and low FGF signals, respectively. Within midrange FGF signals, ERK activity shows bistability, so that it can be in both states.

Consider a newly generated undifferentiated cell at the posterior PSM end. The cell is initially in the up state because of a high FGF signal, and then its relative position shifts anteriorly alongside tail elongation, leading to a gradual FGF decrease. When the cell reaches the bifurcation point (arrow in **Fig 2B**), the cell suddenly inactivates ERK and transits into the down state. Thus, the model generates a step-like distribution of the ERK activity, as observed in the PSM.

### Modeling ERK-mediated somite differentiation

Based on the ERK dynamics, the PSM cells sequentially experienced three distinct states: undifferentiated, intermediate, and differentiated (committed as presumptive somites) (**Fig 2C**). The cells start in the undifferentiated state, characterized by an “up” ERK activity state (**Fig 2B**, *red cells*). Upon ERK inactivation, the cells in the “down” state enter an intermediate state. As a consequence of the up-down ERK activity state transitions, multiple intermediate state cells emerge in the PSM as a cluster (**Fig 2B**, *cyan cells*). Intermediate cells in the cluster collectively differentiate and form a presumptive somite (**Fig 2B**, *grey cells*) after the cluster did not grow (the up-down ERK activity state transition did not occur) within a specific time interval.

As mentioned earlier, cell-cell interaction is incorporated such that intermediate state cells are assumed to suppress ERK activity in neighboring cells. In the fourth term in Eq (2), *χ*_*i*_ indicates the set of neighboring cells of cell *i*, and *S*_*j*_ is the binary value indicating the intermediate state of the neighboring cell *j*. For intermediate state cells (i.e., with a down ERK activity state), *S*_*j*_ = 1; otherwise (i.e., undifferentiated or differentiated states), *S*_*j*_ = 0.

## Results

### Stepwise shift of ERK activity distribution

Using this computational model, we simulated dynamic changes in ERK activity and ERK-mediated somitogenesis (**Fig 2D–2F**). All parameter values used for simulation are listed in **S1 Text**. A sharp ERK activity border was generated at a specific position in the PSM (**Fig 2D**) depending on the ERK activity bifurcation diagram (**Fig 2B**). In addition, because of the continuous FGF gradient movement toward the posterior end and the periodic clock effect on ERK activity, the border regressed in a stepwise manner (**Fig 2D**), during which a group of cells collectively showed an up-down ERK activity transition at almost the same time (**Fig 2E**). Consequently, constant-sized somites were repeatedly formed (50.0 μm) (**Fig 2F**). Consistent with a previous experimental study [22], the somite size is determined by the PSM growth distance within a clock period (**S1 Fig**). Thus, our model successfully demonstrated stepwise ERK activity shift and normal somitogenesis, as observed *in vivo* (**Fig 1**).

### Robustness against noise

**Fig 3:**
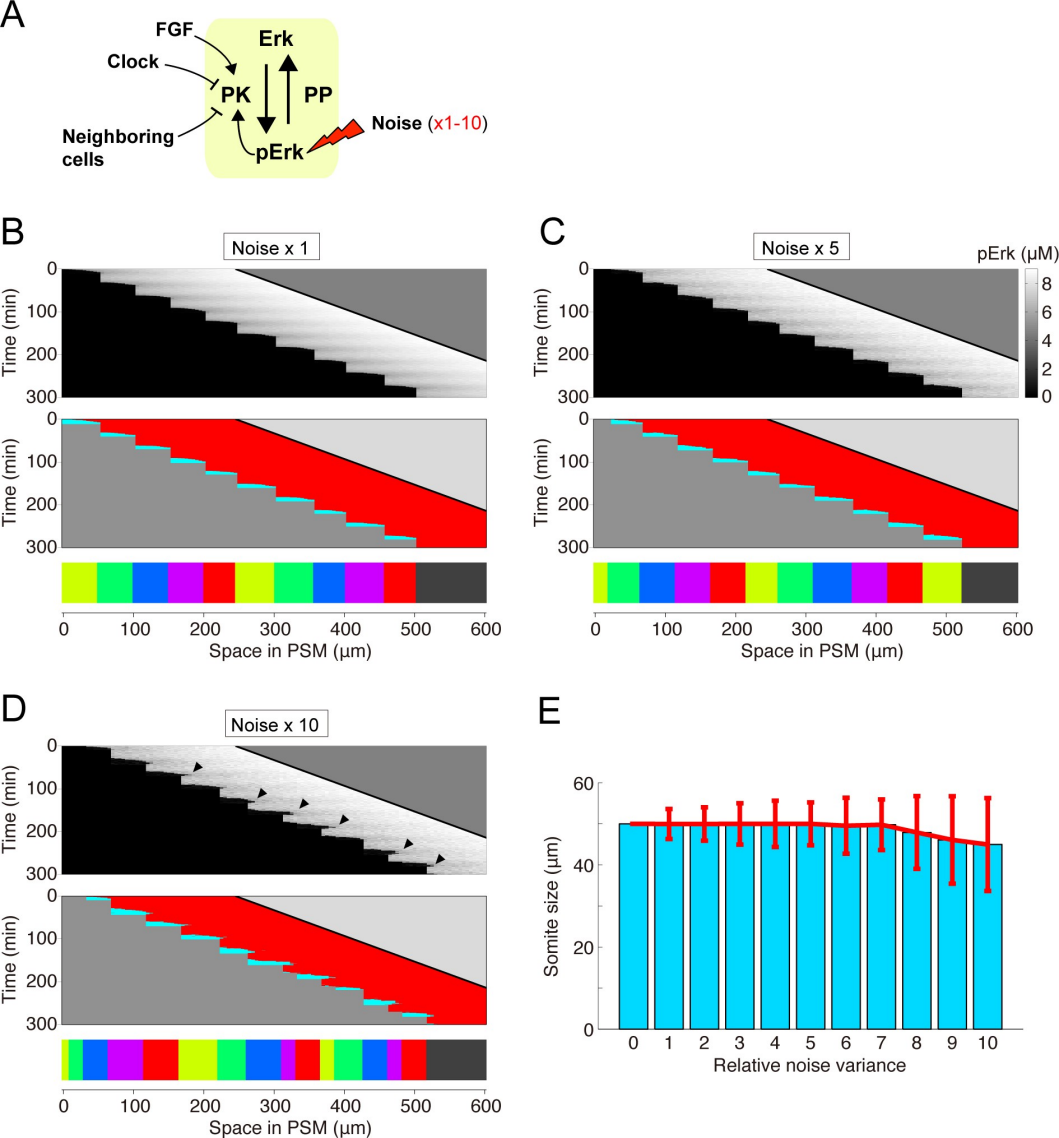
Somite size robustness against reaction noise. **(A)** Simulation settings. Simulations were performed with varying noise variance (i.e., *w* = 0.3–3 in Eq 2). **(B-D)** Representative simulation results of ERK activity (upper panel), state transition (middle panel), and the resultant somites (lower panel) upon noise variance at standard conditions (B) or increased 5- (C) or 10-fold (D). **(E)** Noise effect on somite size. The formation of 200 somites was simulated by changing the noise variance from 0 to 10-fold. Data represent the means and standard deviations.

To explore the effect of intrinsic noise on ERK-mediated somitogenesis, we perturbed this system by adding noise into the model (**Fig 3A**). Even in the presence of the noise, the ERK activity border was generated and moved toward the posterior end in a stepwise manner, resulting in normal somitogenesis (50.0 ± 3.6 μm; C.V. = 0.07) (**Fig 3B**). The system tolerated the noise upon further increase in the noise variance (*w*) over a wide range (at least from 1× to 7×), leading to a stepwise ERK activity shift and normal somite formation (**Fig 3B–3E**). These results therefore indicated that our model recapitulated normal somitogenesis in the presence of intrinsic noise and that this system resisted and was robust against the noise.

### Role of bistability and cell-cell interaction

**Fig 4:**
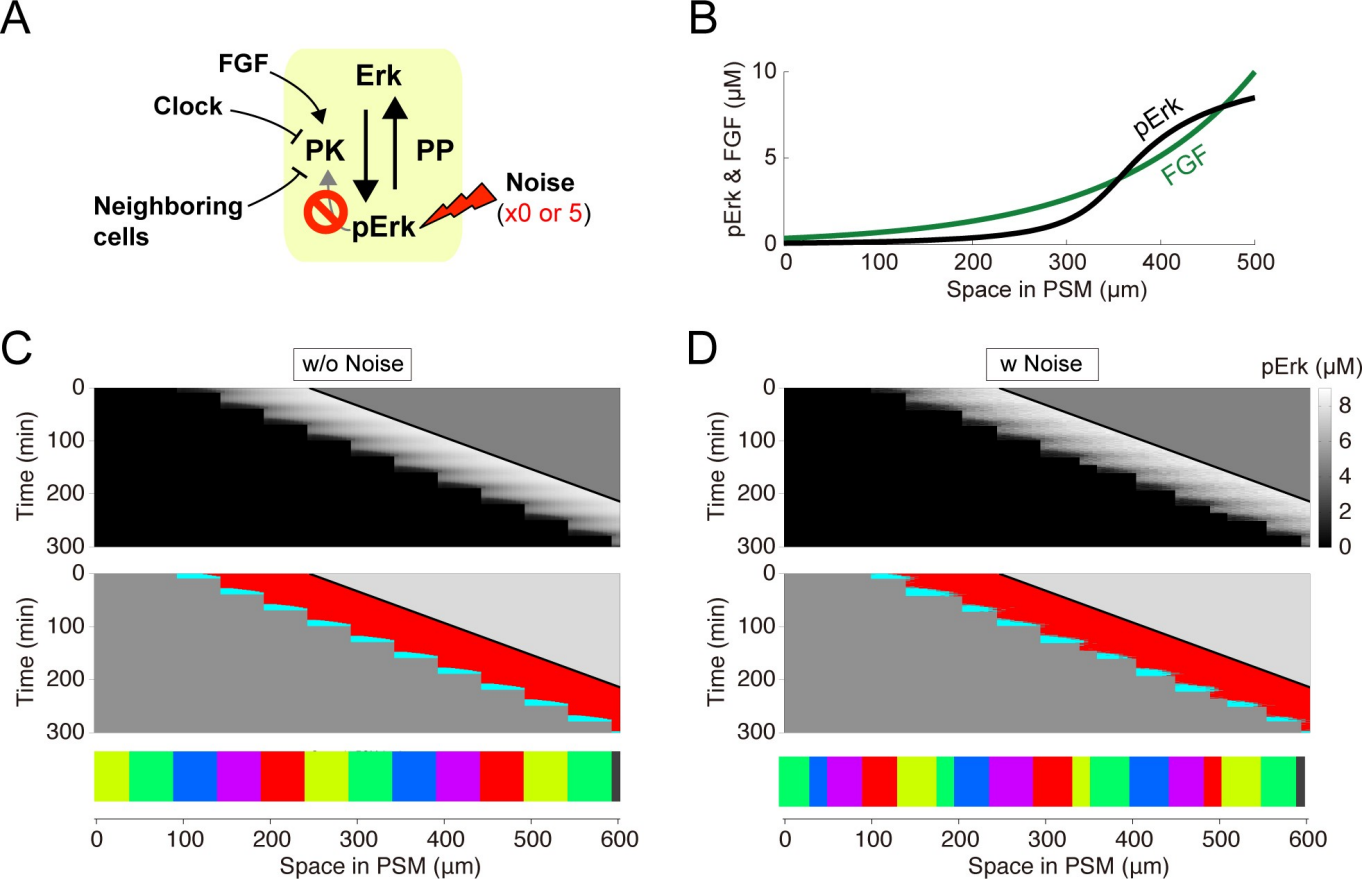
Removing ERK signaling positive feedback erases robustness against noise. **(A)** Simulation settings. Simulations were performed with a 5-fold increase in noise variance upon positive feedback removal (i.e., *w* = 1.5, *a* = 0 in Eq 2). **(B)** Without positive feedback, PSM ERK activity was monotonically distributed (black line). Green line, FGF gradient. **(C)** Somites are regular in the absence of noise (50.0 ± 0 μm; C.V. = 0). **(D)** In the presence of noise, somites become irregular (34.4 ± 12.0 μm; C.V. = 0.35) compared to the same conditions with positive feedback (50.0 ± 5.2 μm; C.V. = 0.10). This result suggests that positive feedback-induced bistability is necessary for somite reproducibility subject to noise.

To investigate how ERK-mediated somitogenesis obtains robustness against noise, we removed each component from our model. Upon removal of the positive feedback regulation of ERK (**Fig 4A**), ERK activity showed a mild gradient within the PSM, rather than a sharp border owing to the lack of bistability (**Fig 4B**). In the absence of noise, the model, which lacks positive feedback regulation, showed a stepwise ERK activity shift and normal somite formation (**Fig 4C**). However, in the presence of noise, the stepwise ERK activity shift was retained, but somitogenesis became abnormal with the co-existence of variably sized somites (**Fig 4D**). These results suggest that the bistable nature of ERK dynamics, resulting from the positive feedback regulation, is required for robust somitogenesis in the presence of noise.

To test the effects of cell-cell interactions on ERK-mediated somitogenesis and its robustness, we removed these from our model (**S2A Fig**). In the absence of noise and cell-cell interactions, our model generated a stepwise ERK activity shift and normal somite formation (**S2B Fig**). However, in the presence of noise, inhomogeneity of ERK activity among the PSM cells increased depending on the noise effect (**S2C–S2E Fig**), and resultant somites showed higher variance than those obtained in the presence of cell-cell interaction (**S2F Fig and Fig 3E**). In addition, we found that the somite size variance was suppressed by strengthening cell-cell interaction (**S3 Fig**). Therefore, cell-cell interactions may play an important role in filtering ERK activity fluctuations caused by noise and in the robustness of ERK-mediated somitogenesis.

Taken together, these results indicate that stepwise ERK activity shifts were observed irrespective of the presence and absence of bistability or cell-cell interaction and thus are solely caused by a combination of FGF gradient movement alongside tail elongation and the cyclic regulation of ERK activity by the clock. On the other hand, both feedback and cell-cell interaction contributed to noise-resistant stepwise ERK activity shifts.

### Role of the segmentation clock

**Fig 5:**
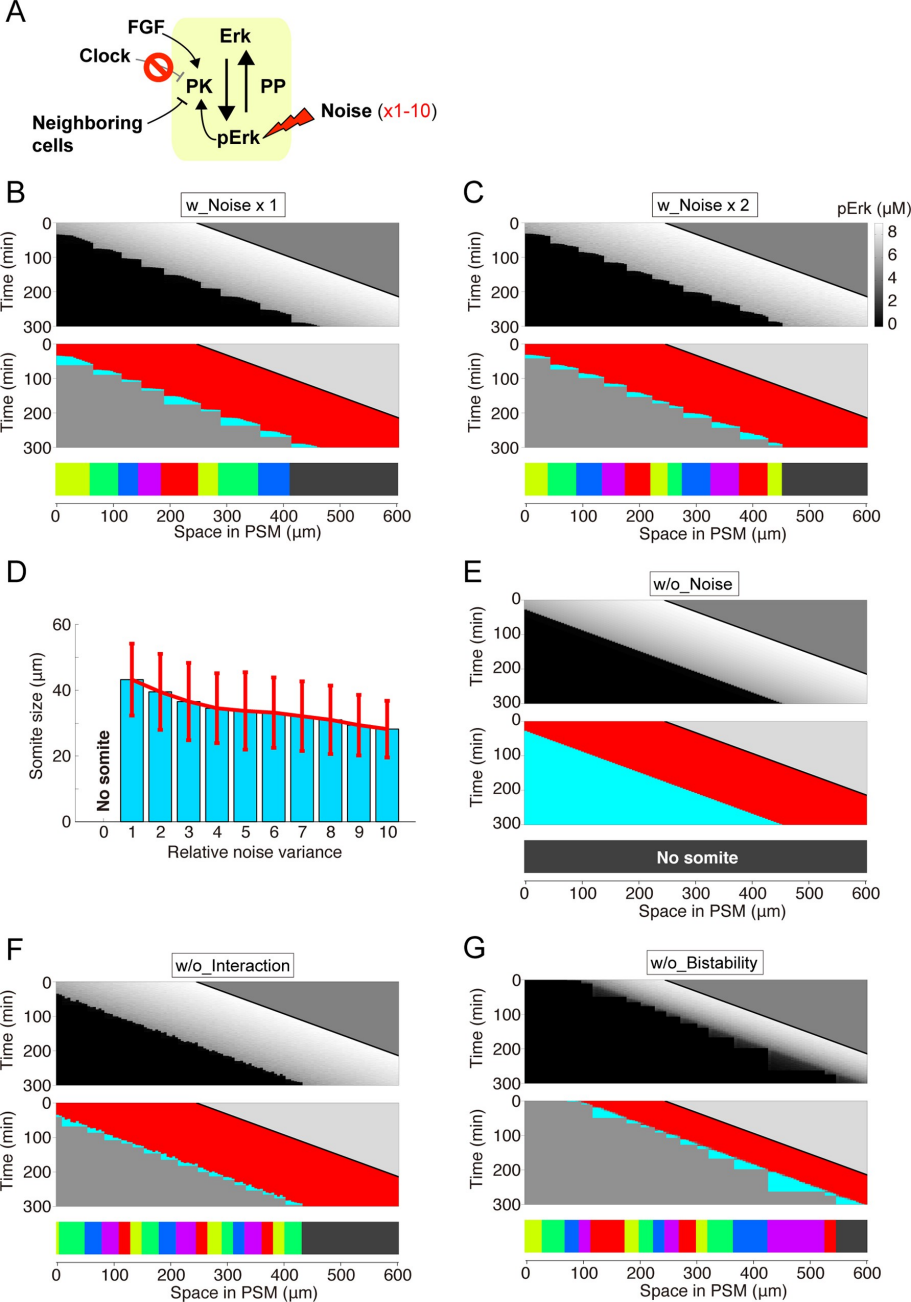
Stepwise ERK activity shift without the clock. **(A)** Simulation settings. Simulations were performed upon clock removal (i.e., *d* = 0 in Eq 2). **(B-C)** Representative simulation results minus the segmentation clock with noise ×1 (B) and ×2 (C) **(D)** Noise effect on somite size without the clock. The formation of 200 somites was simulated by changing the noise variance from 0 to 10-fold. Data represent the means and standard deviations. **(E-G)** Representative simulation results without both clock and noise (E), without both clock and cell-cell interactions (26.2 ± 6.5 μm; C.V. = 0.24), (F) and without both clock and bistability (38.5 ± 19.2 μm; C.V. = 0.50) (G). Neither the stepwise ERK activity shift nor regular somite patterning occurred under these conditions.

Finally, we removed the clock from the model (**Fig 5A**). The ERK activity border alternated between continuous movements and intermittent stops, at which somites were formed (**Fig 5B and 5C**). Thus, the model predicted that a stepwise ERK activity shift still occurred even without the clock, albeit with highly variable shift periodicity, leading to irregularly sized somite formation (**Fig 5D**). The segmental defects in our model were reminiscent of those observed in clock-deficient embryos such as *her1* and *her7* double morphants and/or mutants (**Fig 1C**) [5,11,19]. Simulated somite variability (43.2 ± 10.9 μm; C.V. = 0.25 in Fig 5B) was similar in range to that seen in clock-deficient embryos (61.1 ± 16.9 μm; C.V. = 0.28) (**Fig 1D**). However, these segmental defects were not observed when we removed both the clock and the noise from our model; the ERK activity border continuously moved toward the posterior PSM and no somites formed (**Fig 5E**), suggesting that intrinsic noise is utilized in ERK-mediated somitogenesis in living embryos.

To examine how the clock-independent stepwise ERK activity shift is generated, we removed cell-cell interactions and positive feedback from the clock-less model. Upon removal of cell-cell interactions, the stepwise ERK activity shift disappeared and small irregular somites were formed (**Fig 5F**), as cells independently undergo the ERK activity up-down transition at random times owing to the noise around the specific FGF signal level. Upon removal of positive feedback, which eliminates the bistability of ERK activity, the stepwise ERK activity shift also disappeared (**Fig 5G**).

**Fig 6:**
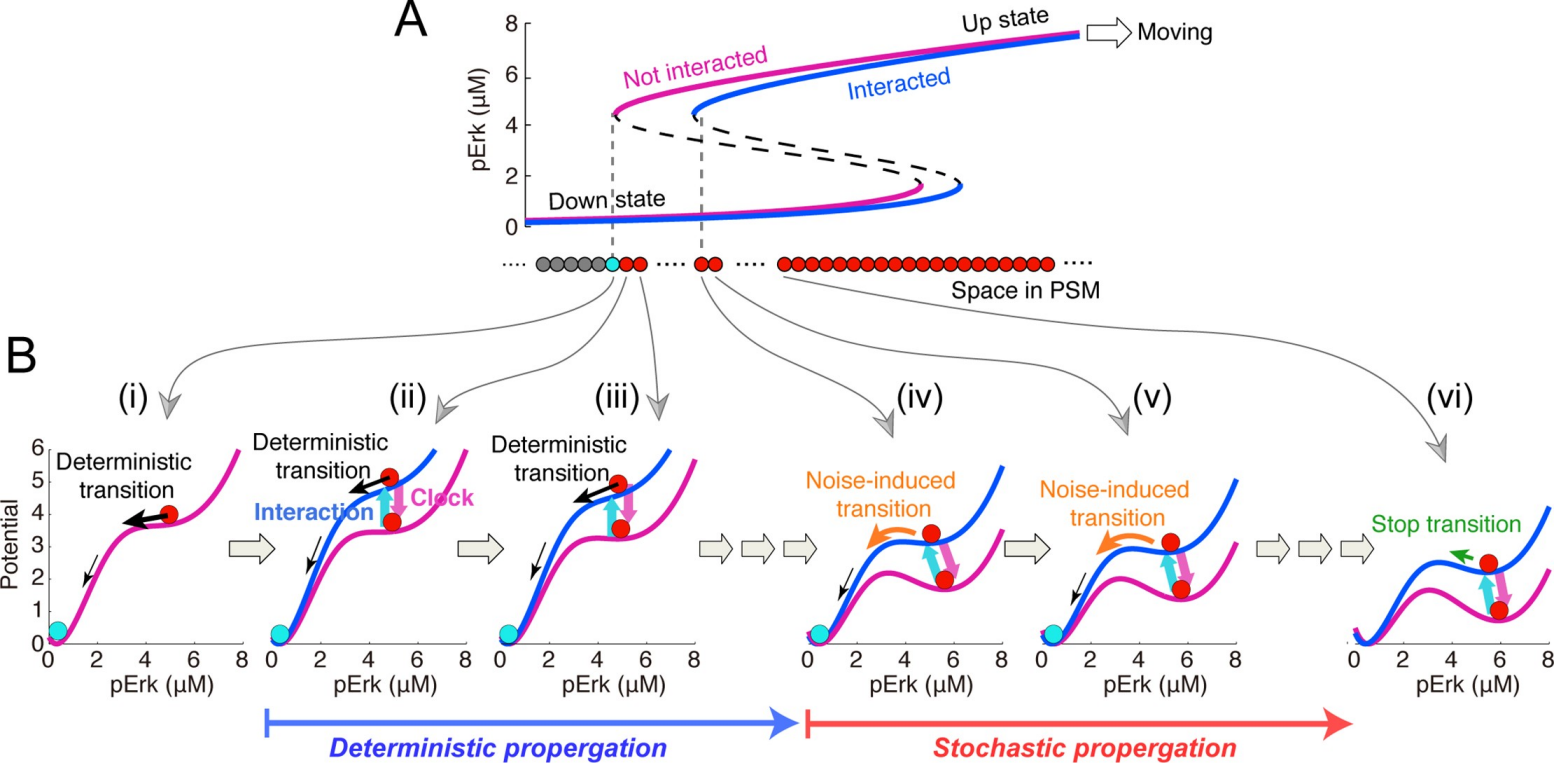
Mechanism of stepwise ERK activity shift. **(A)** Spatial bifurcation diagrams of ERK activity in the absence and presence of interaction with an intermediate cell are depicted by magenta and blue lines, respectively. **(B)** Stochastic dynamics of ERK activity in potential landscape of each cell. Magenta and blue lines indicate potential landscapes when cells are independent and interact with intermediate cells, respectively. Cell (*i*) enters the intermediate state via the up-down transition of ERK activity and then suppresses the ERK activity of cell (*ii*). In cell (*ii*), the stable up state is destabilized by uplifting of the potential landscape by interaction with cell (*i*) and deterministically transits to the down state. After that, the same event happens in cell (*iii*) and continues posteriorly as a deterministic propagation. In cell (*iv*), the up state uplifted by interaction with an anterior neighboring intermediate cell is still stable but can stochastically transit to the down state owing to noise perturbation. The same event could happen in cell (*v*), continuing posteriorly as a stochastic propagation. In cell (*vi*), the potential barrier of the uplifted landscape becomes so high that noise cannot overcome for inducing the up-down transition within the time interval required for somite differentiation from intermediate cells.

Based on these model predictions, the clock-independent stepwise ERK activity shift at irregular timing and distance essentially requires noise, cell-cell interaction, and positive feedback and thus is caused by spatial propagation of noise-induced up-down transition of ERK activity through cell-cell interactions (see **Fig 6**; Discussion for details). This mechanism is different from that of regular stepwise shift in the presence of clock. Most importantly, we concluded that the clock has two distinct roles in somitogenesis: temporal regulation of stepwise ERK shift, and improvement of ERK-mediated somitogenesis developmental reproducibility. In support of this, we also found that the noise-resistant reproducibility of somites increases by amplifying the clock signal (**S4 Fig**).

### Noise-resistant somite reproducibility by clock

**Fig 7:**
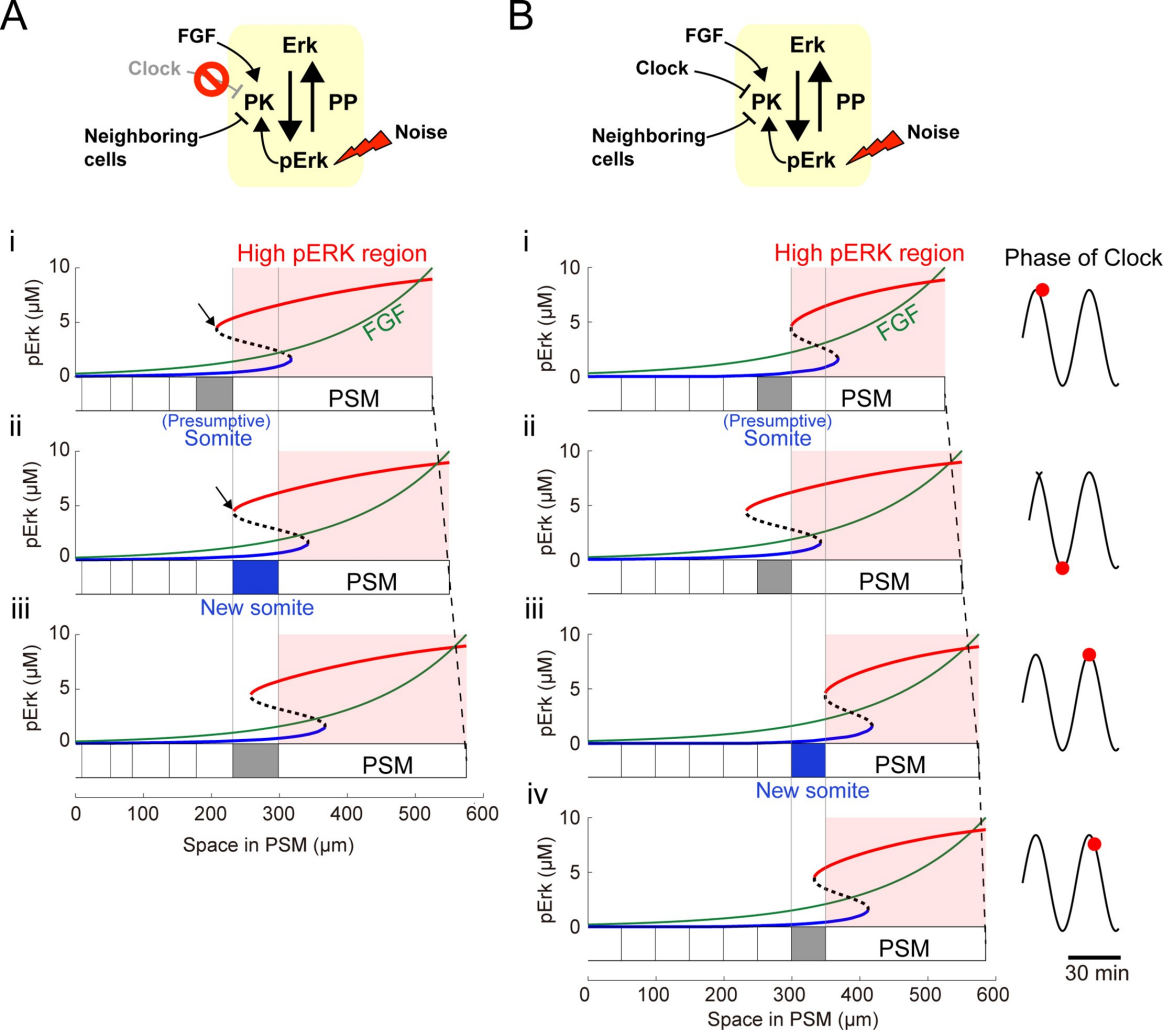
Mechanism of noise-induced irregular somite formation and clock-regulated somite reproducibility. Schematic representation of simulation conditions (upper panels). **(A)** Spontaneous irregular somite formation without the segmentation clock. (*i*) After a group of PSM cells forms the presumptive somite, the ERK activity bifurcation point localizes to the anterior presumptive somite (black arrow). (*ii*) Depending on FGF gradient posterior movement, the bifurcation point reaches the posterior presumptive somite border. Then, ERK activity rapidly transitions from the up to down state in undifferentiated cells, which enter the intermediate state. Cell-cell interaction propagates the ERK activity up-down state transition toward the posterior PSM in a chain-like manner (blue area), stopping at an irregular distance owing to the inevitable stochasticity. (*iii*) The process indicated in (*i*) is repeated after (ii). **(B)** Precise somite formation induced by the segmentation clock. (*i*) At peak clock phase, the clock-dependent ERK activity inhibition also peaks, drastically shifting the bifurcation diagram toward the posterior end [compare with (A-*i*)] and forming a new presumptive somite. (*ii*) At the clock phase midpoint, the bifurcation diagram reverts to the anterior end [compare with (A-*ii*)], because the segmentation clock cannot inhibit ERK activity therein. (*iii*) Upon clock phase return, the bifurcation diagram shape gradually shifts toward the posterior end [compare with (B-*i*)]. During this phase, a PSM cell group transitions from the up to down ERK activity state, resulting from temporal oscillation and spatial bifurcation diagram shift, due mainly to the segmentation clock and PSM growth, respectively. Also, the up-to-down ERK activity transition tends to propagate toward the posterior end owing to the inevitable stochasticity, which may lead to somite irregularities as with segmentation clock loss [(A-*ii*) and Fig 1C]. (*iv*) Immediately thereafter, the propagation terminates at a fixed distance, because the bifurcation diagram starts to shift toward the anterior end [compare with (B-*iii*)].

We investigated how somite reproducibility is generated against the noise. In the absence of cell-cell interactions and the clock, individual cell ERK activity levels differed owing to the noise (**Fig 5F**). Thus, ERK activity up-down transitional timing inevitably differed among cells, leading to stepwise ERK activity shift failure. Adding cell-cell interactions locally coupled ERK activity among the neighboring cells, resulting in reduced ERK activity homogeneity among neighboring cells (**Fig 5B**). Because the ERK activity shift timing differed owing to noise effects, the resultant somite size was highly variable (**Fig 5D**). Therefore, our simulations suggest that cell-cell interactions contribute to filtering ERK activity homogeneities among neighboring cells and that the filtering property adversely converts the noise effect within individual cells into ERK activity spatial heterogeneity (see **Fig 6** and **Fig 7A**; Discussion for details).

Adding the clock into our model reduced ERK activity spatial heterogeneity, which is caused by noise and cell-cell interactions, leading to reproducible constant-sized somite formation (**Fig 3B**). To understand its mechanism, segmentation clock effects on the PSM ERK activity bifurcation diagram were investigated. Because of the clock, the ERK activity bifurcation diagram cyclically shifted back-and-forth in the PSM (see **Fig 7B**; Discussion for details). After each clock cycle, the bifurcation diagram shifted to the posterior end owing to PSM elongation. Thus, the ERK activity up-down transition was precisely restricted to a specific time and within specific space intervals. Therefore, these results suggest that the segmentation clock both provides temporal information on somite formation as in the classical CW model and reduces ERK activity spatial heterogeneity against the noise, representing a previously unidentified role of the segmentation clock.

## Discussion

The mechanisms underlying the precision and reproducibility of developmental events within embryos in multicellular organisms despite cell-to-cell variations in mRNA and protein levels caused by stochastic gene expression [23] remain unclear. Through computational modeling, we clarified the mechanism by which high reproducibility among somites is achieved. We also proposed a novel concept wherein temporal regulation of the global signal; i.e., the segmentation clock, reduces the noise effect to enhance developmental reproducibility.

### Model prediction

**Fig 8:**
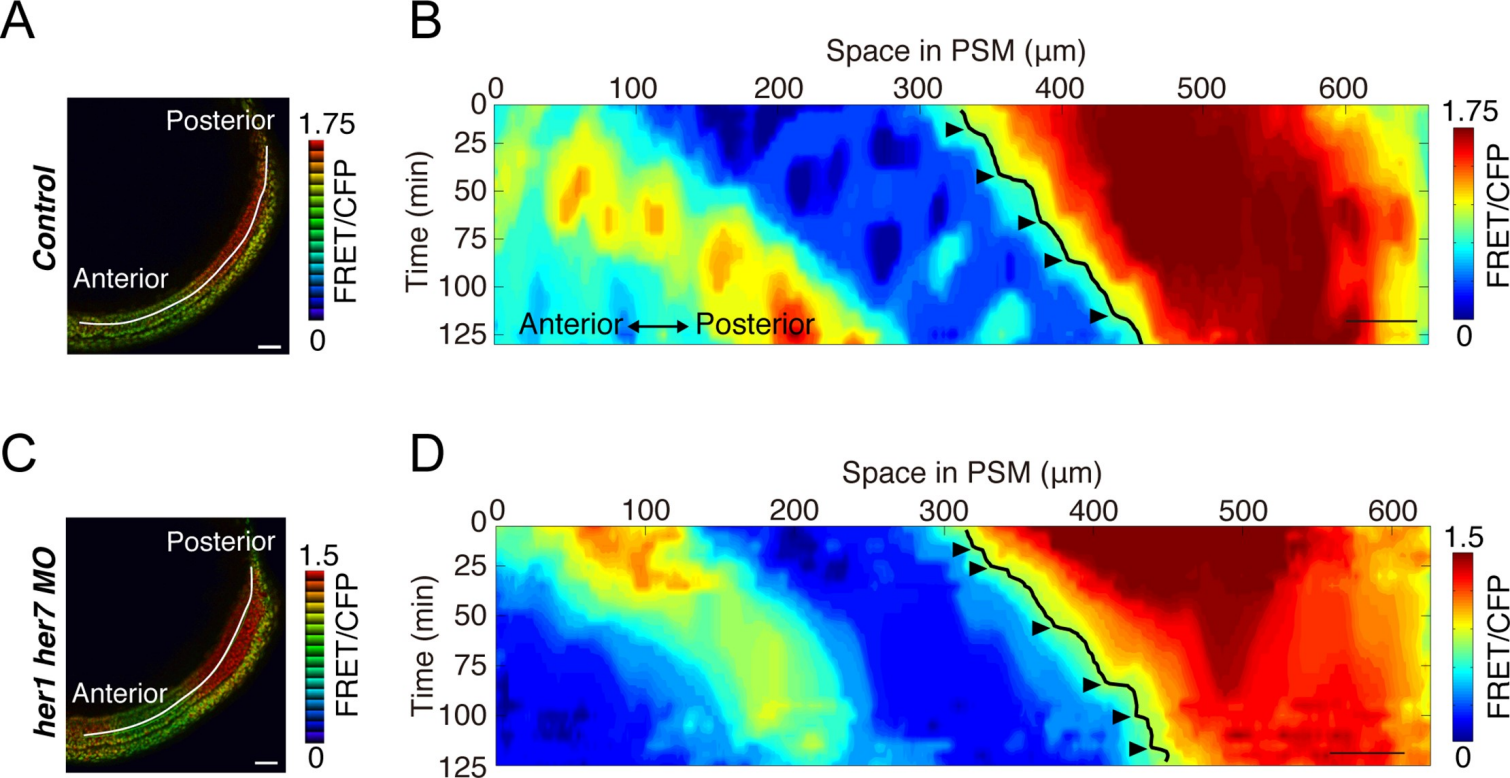
Stepwise ERK activity shifts in control and clock-deficient zebrafish embryos. (A, C) FRET-based ERK biosensor mRNAs were injected into control (A) and clock-deficient zebrafish embryos (C). ERK activity was calculated by FRET/CFP ratio in embryos at the 8-somite stage and visualized by intensity-modulated display mode. A line of interest for kymograph analysis is depicted. Scale bar, 50 μm. (B, D) Kymographs of the control and clock-deficient embryos. ERK activity is shown by the heat map. Black lines indicate the ERK activity borders, which show the stepwise shift at regular timing in control embryo (B) and at irregular timing in clock-deficient embryo (D). Scale bar, 50 μm. This figure is modified from our recent paper [24].

The CW model predicted that clock removal impeded somite individualization, as seen in simulation without clock and noise (**Fig 5E**). This contradicted clock gene loss-of-function phenotypes wherein irregularly-sized somites are still formed. In contrast, our model has an ability to generate such somites, predicting that stepwise ERK activity shift still occurred at irregular timing with irregular distances in clock-deficient embryos (**Fig 5B and 5C**). This may explain why a stepwise ERK activity shift could not be observed in clock-deficient embryos in our previous static analysis based on collected ERK activity snapshots from different embryos [5]. We recently performed *in vivo* imaging of ERK activity in zebrafish embryos using a FRET-based biosensor of ERK [24] and confirmed our model prediction that stepwise ERK activity shifts happen in control and clock-deficient embryos in regular and irregular manners, respectively (**Fig 8**). Therefore, our model was experimentally validated.

### Comparison with previous models

The CW model, originally proposed as a conceptual model [9], was revised upon clock and wavefront molecular bases discovery [25–27]. Several theoretical models have also been proposed to explain the different aspects of somitogenesis, including the mechanism of PSM cell oscillatory gene expression [27,28], clock synchronization among cells [29–32], and PSM spatial oscillatory patterning [33]. Although two models addressed the noise effect on clock synchronization [29,32], none addressed that on somite commitment. Thus, our model is the first to satisfactorily explain how somite reproducibility is maintained subject to noise.

### Validity of model components

Our model exhibits biological relevance in terms of the intrinsic noise, ERK signaling bistability, cell-cell interactions, and the segmentation clock.

#### Intrinsic noise

In our model, added noise perturbed ERK activity. Physically, chemical reactions happen stochastically, subjecting molecular concentrations to random fluctuations when molecules are present in low copy numbers [1–3]. Synthetic biological studies on bacteria and yeast demonstrate the inevitability of reaction noise in gene expression regulation [1,3]. Although it is difficult to identify the noise source and manipulate its intensity in the developing embryo, we speculate that it originates from intracellular reaction stochastic properties within single cells.

#### ERK signaling bistability

In a concept of canalization proposed by Waddington [34], the cell state follows a downward slope to reach a stable point associated with cell fate transition. In a current interpretation, cell fate transitions are defined by stable gene expression patterns. Accordingly, previous somitogenesis models incorporated two stable states (i.e., bistability, which is associated with cell fate transition during somite individualization) [9,25]. Because we previously observed up and down PSM ERK activity states (**Fig 1G**), and as *Xenopus* oocyte ERK activity exhibits bistability, our model incorporated ERK signaling bistability regulated by positive feedback [35].

#### Cell-cell interaction

Somite formation is a type of collective cell fate transition in a population of cells, suggesting that cell-cell interactions are involved in somite formation. It has been reported that the spontaneous formation of somite-like structures in the absence of the clock requires cell-cell interactions [21]; moreover, PSM cell ERK activity levels are similar to those of adjacent PSM cells [5,24]. Based on these findings, our model assumed cell-cell interactions, such that cells in the ERK activity down state suppress neighboring cell ERK activity (**Fig 2A**).

Although the molecules responsible for these cell-cell interactions have not been identified, many cell adhesion molecules, including cadherins, proto-cadherins, and connexins, are expressed in the PSM during somitogenesis (data retrieved from the Zebrafish Information Network, http://zfin.org/, December 12, 2016) and may contribute to the coupling of ERK activity among cells. Indeed, cadherins and connexins were reported to suppress ERK activity [10,36], and so those molecules should be investigated in future studies.

#### Segmentation clock

No direct evidence supports the hypothesis that clock genes (*her1* and *her7*) temporally modulate zebrafish ERK activity. However, negative ERK regulator (e.g., *sprouty* and *dusp*) mRNA expression oscillates in the mouse and chick [10]. Thus, we considered that the segmentation clock temporally regulates PSM ERK activity. In the model, we tentatively assumed a negative clock effect on ERK activity (**Fig 2A**). However, the model reproduced a stepwise ERK activity shift regardless of clock effect direction (**Fig 2D and S5A Fig**), suggesting the relevance of clock periodicity to signaling but not effect direction.

We assumed that clock signal is completely deleted in embryos co-injected with *her1* and *her7* morpholinos (**Fig 1C and Fig 8C**), because irregular somites are generated as observed in *her1/her7*-double KO zebrafish embryos [19]. However, there is a possibility that *her1* and *her7* are still functional as a clock with reduced amplitude induced by the morpholinos. We thus decreased the clock amplitude in the model and confirmed that irregular stepwise shift of ERK activity happens as seen in clock-deficient cases (**S5B Fig**), indicating that it does not matter whether the clock effect is maintained. We also showed that somite size variability increases with a decrease in the clock amplitude (**S4E Fig**).

The clock amplitude was constant in the model, while this is not necessarily the case in reality. We thus incorporated temporal fluctuation of the clock amplitude into the model. We found that somite sizes largely vary based on clock amplitude fluctuation (**S5C Fig**). In contrast, somites showed resistance to ERK activity noise over a wide range of clock amplitudes, as long as it was kept constant above a certain threshold (**S4E Fig**). Therefore, this suggests that the constancy of the clock amplitude, but not amplitude itself, is important for somite reproducibility.

#### Intermediate state and time interval for somite differentiation

In the posterior PSM cells, undifferentiated states are maintained while ERK is activated by FGF [18]. Upon the inactivation of ERK, the cells are not ready to instantaneously differentiate into a somite but rather a specified time interval is required for being ready to differentiate through multi-step processes, including transcription and translation of essential factors and protein transport and degradation. We thus modeled the intermediate state and the time interval for somite differentiation. In the model, a change in the time interval did not affect somite formation in the presence of the clock, whereas it regulated the mean somite size in the absence of the clock (**S6 Fig**).

### Mechanism of stepwise ERK activity shift

To understand the mechanism of the irregular stepwise ERK activity shift in the absence of the clock, we rewrote Eq (1) as

$$\frac{d[pErk_{i}]}{dt}=-\frac{d\phi \left(\left[pErk_{i}\right]\right)}{d[pErk_{i}]}+\sqrt{w}\xi_{i},$$ (5)

where *ϕ*([*pErk*_*i*_]) indicates the potential landscape. Here, we consider a specific moment when the moving bifurcation point reaches the most anterior undifferentiated cell (cyan cell in **Fig 6A**). The cell has a single-well potential with a stable down state of ERK activity so that the up state transits to the down state, entering the intermediate state (**Fig 6B-*i***). Then, the intermediate cell starts to suppress the neighboring cell, which makes a double-well potential change to a single-well potential with a stable down state of ERK activity, and then the ERK activity deterministically transits to the down state (intermediate state) (**Fig 6B-*ii***). Such up-down transitions deterministically propagate toward the posterior end (**Fig 6B-*iii***). After the deterministic propagation progresses several steps, a double-well potential is maintained even with ERK suppression by the neighboring cell (**Fig 6B-*iv***). In this situation, noise can induce transition from the up to down state (intermediate state) [2]. As up-down transitions stochastically propagate posteriorly (**Fig 6B-*v***), the potential barrier from the up to down state increases, and finally the state transition cannot happen within the specific time interval required for somite differentiation. Therefore, the stepwise ERK activity shift results from both deterministic and stochastic propagations of the up-down ERK activity transition via cell-cell interactions.

According to this view (**Fig 6**), it can be speculated that the increase in noise intensity produces larger somites, because noise enhances the stepwise ERK activity shift through stochastic propagation. However, we have obtained opposite simulations results that show that average somite size decreases with an increase in noise intensity, both in the presence and absence of clock (**Fig 3E and Fig 5D**). How can we understand these facts? In the presence of clock, the ERK activity border sometimes overshoots the correct location toward the posterior end due to the enhanced stochastic propagation and comes back toward the anterior end due to the clock effect (arrowheads in **Fig 3D**), which generates small somites. In contrast, in the absence of clock, the ERK activity border shifts toward the posterior much faster than the PSM growth speed due to the enhanced stochastic propagation, and then the potential barrier of up-down ERK activity transition quickly increases, which halts stochastic propagation after a short distance.

### Clock-independent and -dependent somite formation mechanism

Finally, we proposed mechanisms for clock-independent irregular and clock-dependent regular somite formation from the point of view of a dynamic system subject to noise. Our model predicted irregular ERK activity border shifting in clock-deficient embryos (**Fig 5B and 5C**). Here, we depicted a spatiotemporal bifurcation diagram within the growing PSM (**Fig 7A**). A newly generated cell at the posterior PSM end is exposed to a high FGF gradient, which activates ERK (**Fig 7A**, also see **Fig 2B**). The cell with the up ERK activity state then aligns toward the anterior PSM alongside tail elongation, leading to a gradual FGF gradient decrease. Owing to ERK signaling bistability, ERK remains activated; when the cell reaches a specific point in the bifurcation diagram (black arrow in **Fig 7A-*ii***), ERK suddenly inactivates. This cell then starts to interact with and induce neighboring cells to undergo up-down ERK activity transition, which propagates toward the posterior PSM. Owing to the inevitable stochasticity, this propagation progresses stochastically and terminates if the up-down ERK activity state transition does not happen within a specific time interval (**Fig 7A-*ii***, *blue region*). Thus, the ERK activity border shifts an irregular distance, eventually leading to irregularly sized somite formation.

Given the clock-independent mechanism of irregular somite formation described above, we investigated the role of the segmentation clock in reducing the noise effect on somite reproducibility. With clock present, the ERK activity bifurcation diagram cyclically changed depending on the clock phase (**Fig 7B**). This may explain how the regular somite pattern is generated against the inevitable stochasticity. Within a specific time interval (before peak clock oscillation), ERK is inactivated in an anterior PSM cell cohort (**Fig 7B-*iii***). At this time, the ERK activity up-down transition starts to propagate toward the posterior PSM, as seen upon segmentation clock loss (**Fig 7A**). Immediately thereafter (after peak clock oscillation), however, the bifurcation diagram shifts toward the anterior end, leading to stochastic propagation termination at a fixed distance (**Fig 7B-*iv***). Accordingly, uniformly sized somites are periodically generated.

## Supporting information

S1 Text Parameter setting. (DOCX)

S1 Fig Somite formation with different PSM growth speeds and clock periods. Simulation results under various conditions without noise: control condition (A), decreased clock period (B), increased clock period (C), decreased PSM growth speed (D) and increased PSM growth speed (E).
(TIF)

S2 Fig ERK-mediated somitogenesis without cell-cell interactions. **(A)** Simulation settings. Simulations were performed for cell-cell interaction exclusion (i.e., *d* = 0 in Eq 2). (**B-D**) Representative simulation results when the noise variance was set to standard conditions (B) and increased 5- (C) or 10-fold (D). **(E)** Effect of noise on somite size. The formation of 200 somites was simulated by changing the noise variance from 0 to 10-fold. The data represent the means and standard deviations.
(TIF)

S3 Fig Effect of cell-cell interaction on ERK-mediated somitogenesis. **(A)** Simulation settings. Simulations were performed with a 5-fold increase in noise intensity by changing cell-cell interaction strength (i.e., *w* = 1.5, *d* = 0~2 in Eq 2). (**B-D**) Representative simulation results when the cell-cell interaction strength was decreased 0.2-fold (B), under standard conditions (C), and increased 2-fold (D). **(E)** Effect of noise on somite size. The formation of 200 somites was simulated by changing the interaction strength from 0 to 2-fold. The data represent the means and standard deviations.
(TIF)

S4 Fig Effect of clock amplitude on ERK-mediated somitogenesis. **(A)** Simulation settings. Simulations were performed with 5-fold increase in noise intensity by changing clock amplitude (i.e., *w* = 1.5, *c* = 0~2 in Eq 2). (**B-D**) Representative simulation results when the clock amplitude was decreased 0.2-fold (B), under standard conditions (C), and increased 2-fold (D). **(E)** Effect of noise on somite size. The formation of 200 somites was simulated by changing the clock amplitude from 0 to 2-fold. The data represent the means and standard deviations.
(TIF)

S5 Fig Positive clock effect on ERK activity, reduced clock amplitude, and temporal fluctuation of clock amplitude. **(A)** Simulation result with a positive clock effect (i.e., *c* = −0.2 in Eq 2). Note that somites are normally formed, even when ERK is positively regulated by the clock. **(B)** Simulation result with a reduced clock amplitude (i.e., *c* = 0.02 in Eq 2). Note that somites are irregularly formed as seen in a clock-deficient embryo. **(C)** Simulation result with fluctuation of clock amplitude. Temporal change in clock amplitude was generated by Ornstein-Uhlenbeck process (inset).
(TIF)

S6 Fig Effect of required interval for differentiation from intermediate to differentiated states. Simulations were performed with a 5-fold increase in noise intensity by changing the time interval for somite differentiation in both the presence and absence of the clock (i.e., *w* = 1.5, *c* = 0 or 1 in Eq 2). (**A-C**) Representative simulation results in the presence of the clock when the time interval decreased 0.5-fold (A), under standard conditions (B), and increased 2-fold (C). (**D-F**) Representative simulation results in the absence of the clock when the time interval decreased 0.5-fold (D), under standard conditions (E), and increased 2-fold (F).
(TIF)
