# Supplementary material for: Noise-resistant developmental reproducibility in vertebrate somite formation
Source: PLoS Comput Biol. 2019 Feb 4;15(2):e1006579. doi: 10.1371/journal.pcbi.1006579 (PMC6361423; doi:10.1371/journal.pcbi.1006579)
Supplement: S1 Text — (DOCX) [file pcbi.1006579.s001.docx]

**S1 Text** Parameter setting

The parameters used in the model were as follows: *FGF_tail_* = 4 (μM), 1/*k_d_* = 1.5 (h), *v* = 100 (μm/h), *ERK_tot_* = 10 (μM), *kcat_PK_* = 10 (s^−1^), *kcat_PP_* = 10 (s^−1^), *Km_PK_* = 5 (μM), *Km_PP_* = 0.7 (μM), *PP* = 0.5 (μM), *K* = 3 (μM), *h* = 4, *w* = 0.3, *T* = 30 (min), *a* = 0.7, *b* = 0.5, *c* = 0.05, and *d* = 0.2. For simulation, the pERK threshold between undifferentiated and intermediate states was set to 3 μM. The time interval required for the intermediate to differentiated state transition was set at 240 s. Cell length was 5 μm. These values were selected based on three criteria as follows: (1) FGF gradient formed in the PSM as shown in **Fig. 2B** (for *FGF_tail_*, *k_d_*, and *v*), (2) PSM pERK exhibited a bistable bifurcation diagram depending on the FGF gradient as shown in Fig. 1B (for *ERK_tot_*, *kcat_PK_*, *kcat_PP_*, *Km_PK_*, *Km_PP_*, *PP*, *K*, *h*, *a*, *b*), and (3) segmentation clock cyclically changed the bifurcation diagram as shown in **Fig. 7B** (from *i* to *iii*). Under conditions that satisfied these criteria, the basic behaviors shown in **Fig. 2D-F** and **5B** were robustly reproduced.
