# Supplementary figures and images for: Noise-resistant developmental reproducibility in vertebrate somite formation
Source: PLoS Comput Biol. 2019 Feb 4;15(2):e1006579. doi: 10.1371/journal.pcbi.1006579 (PMC6361423; doi:10.1371/journal.pcbi.1006579)

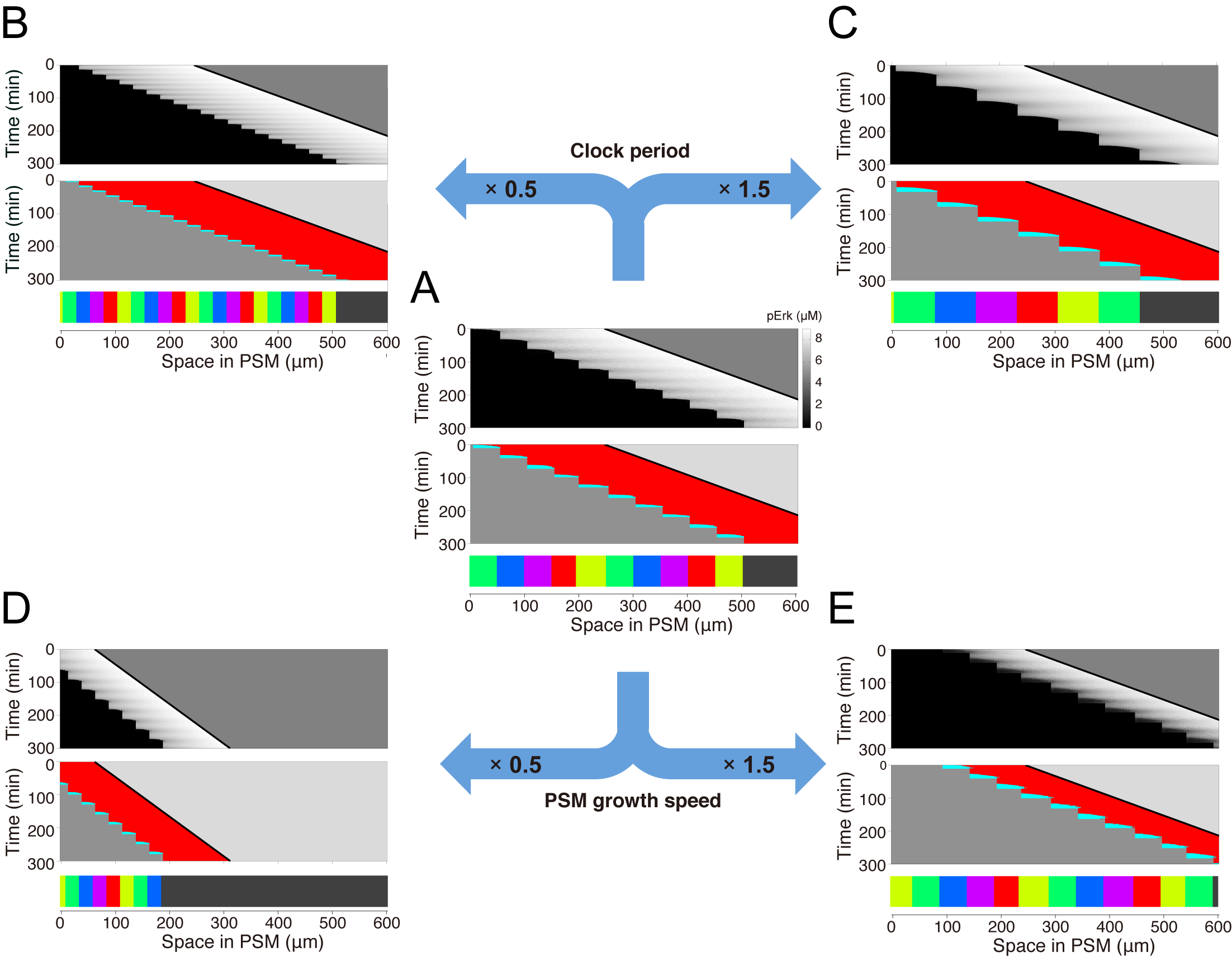

Supplement: S1 Fig — Simulation results under various conditions without noise: control condition (A), decreased clock period (B), increased clock period (C), decreased PSM growth speed (D) and increased PSM growth speed (E). (TIF) [file pcbi.1006579.s002.tif]

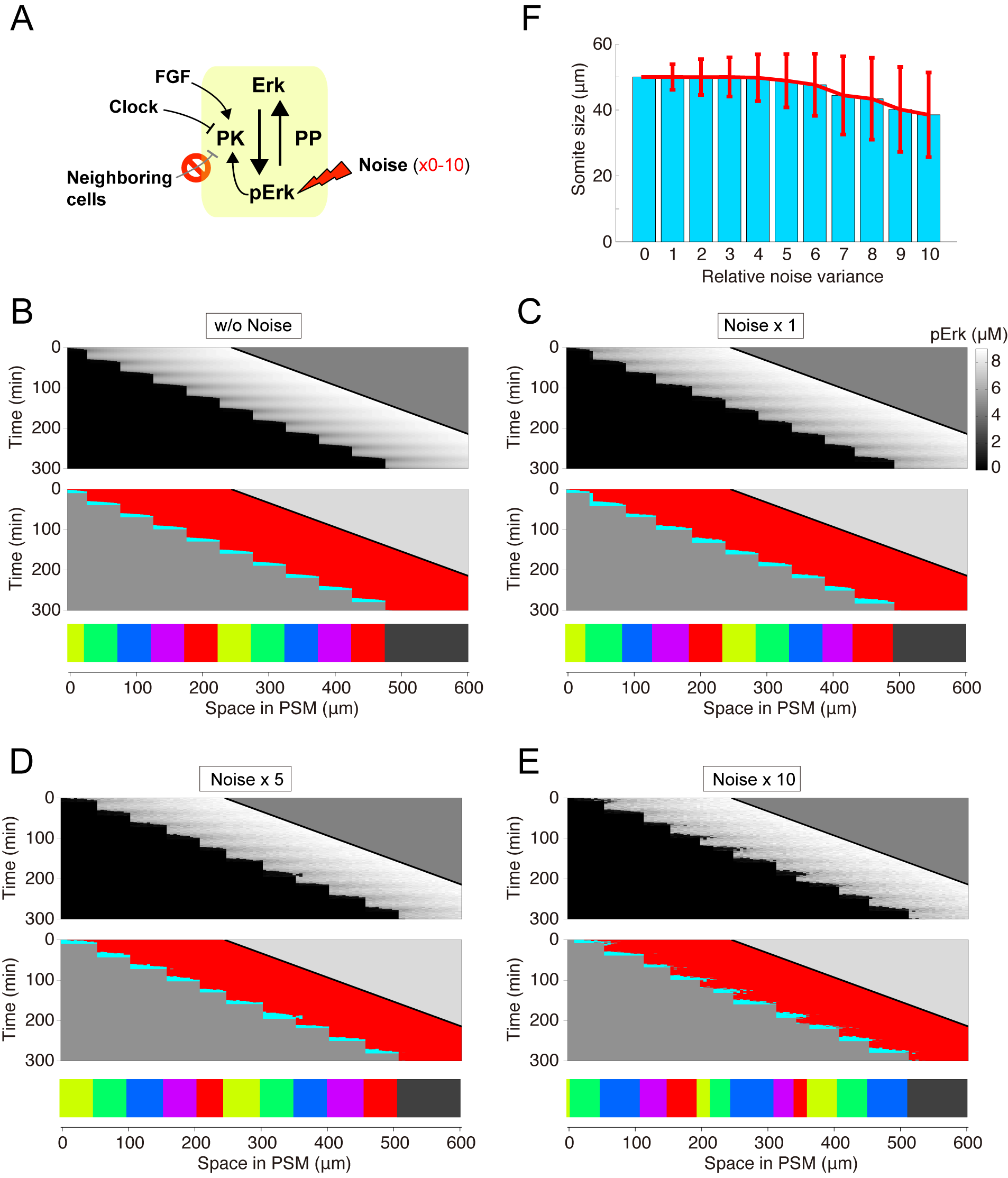

Supplement: S2 Fig — (A) Simulation settings. Simulations were performed for cell-cell interaction exclusion (i.e., d = 0 in Eq 2). (B-D) Representative simulation results when the noise variance was set to standard conditions (B) and increased 5- (C) or 10-fold (D). (E) Effect of noise on somite size. The formation of 200 somites was simulated by changing the noise variance from 0 to 10-fold. The data represent the means and standard deviations. (TIF) [file pcbi.1006579.s003.tif]

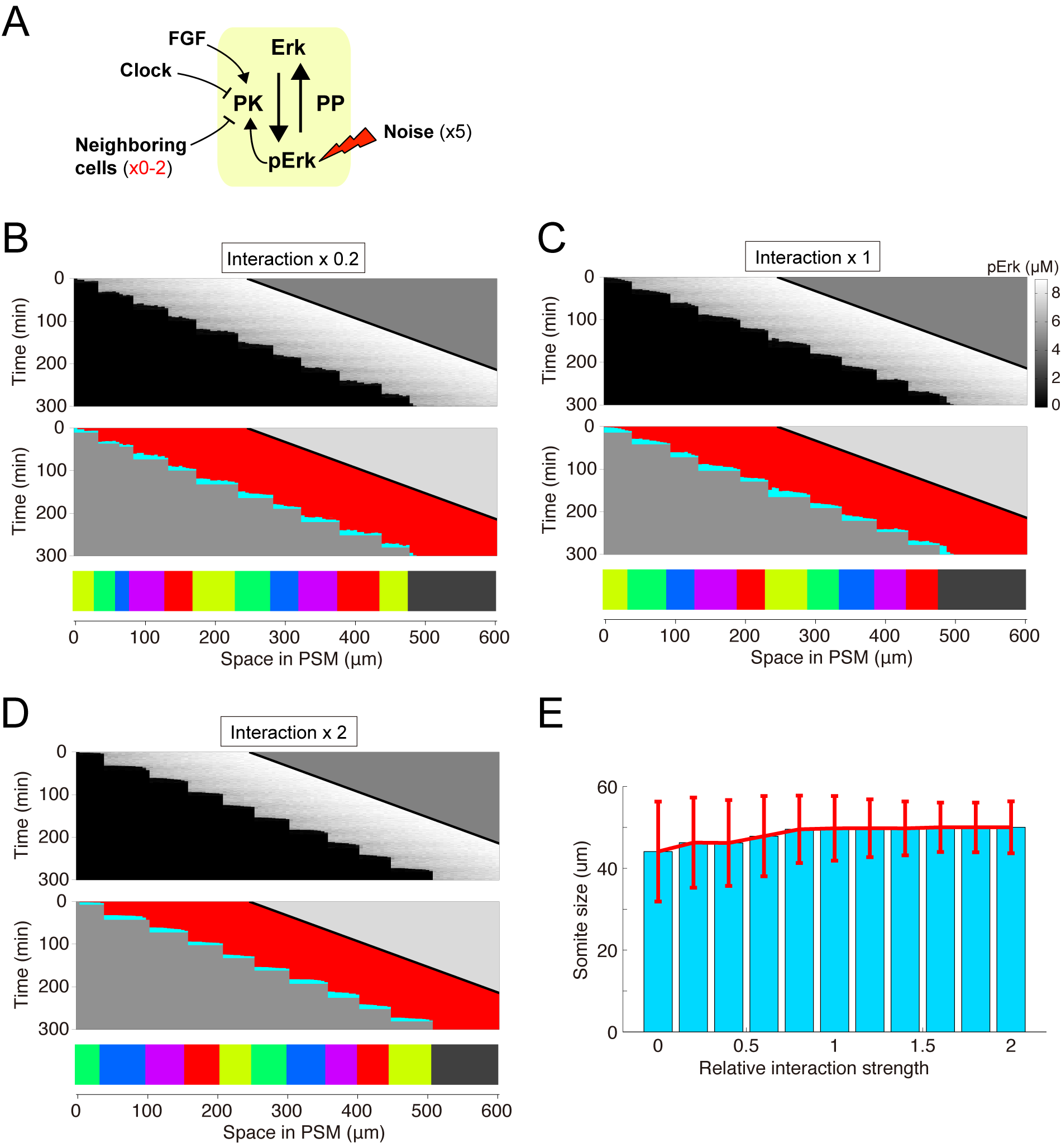

Supplement: S3 Fig — (A) Simulation settings. Simulations were performed with a 5-fold increase in noise intensity by changing cell-cell interaction strength (i.e., w = 1.5, d = 0~2 in Eq 2). (B-D) Representative simulation results when the cell-cell interaction strength was decreased 0.2-fold (B), under standard conditions (C), and increased 2-fold (D). (E) Effect of noise on somite size. The formation of 200 somites was simulated by changing the interaction strength from 0 to 2-fold. The data represent the means and standard deviations. (TIF) [file pcbi.1006579.s004.tif]

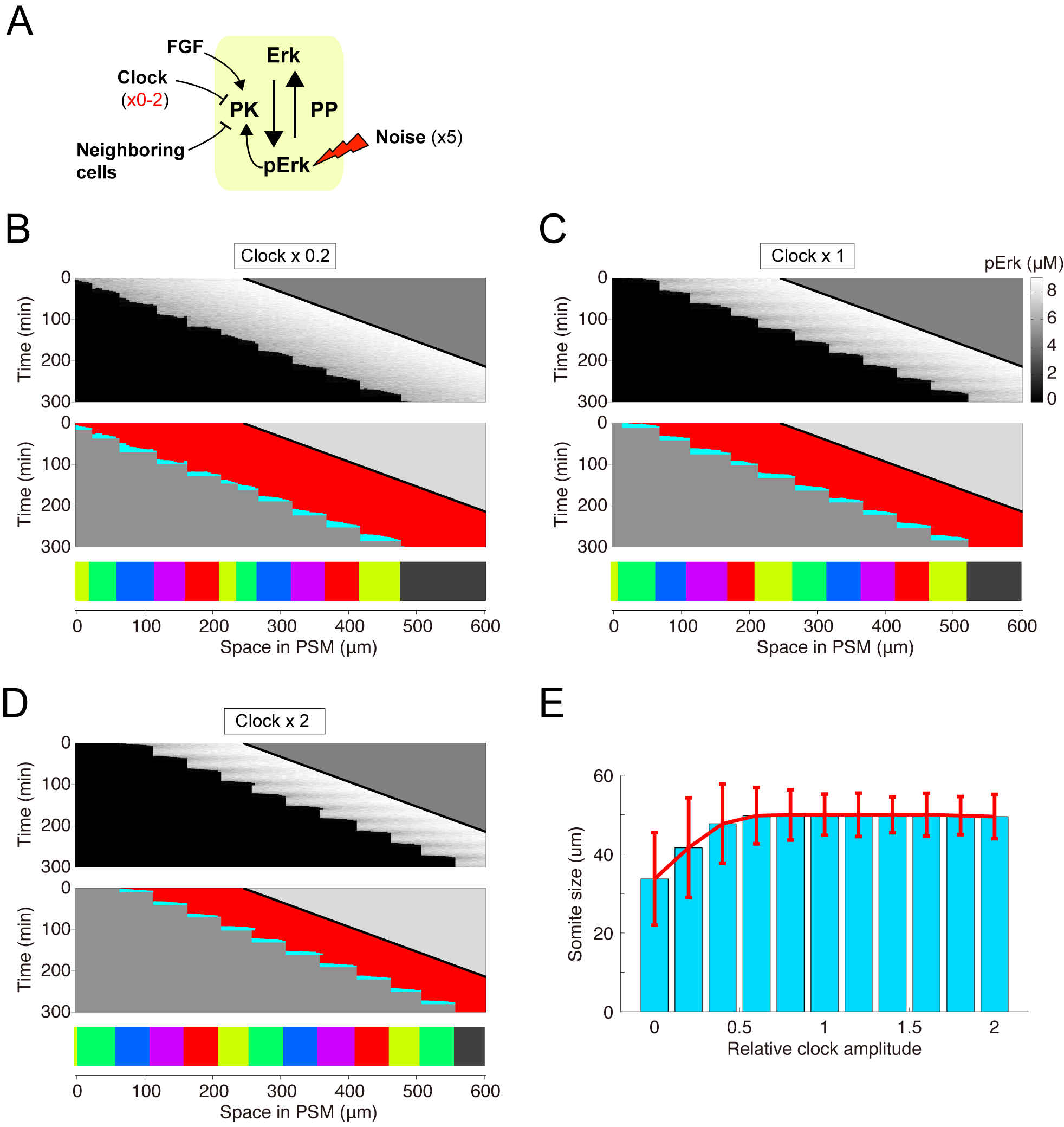

Supplement: S4 Fig — (A) Simulation settings. Simulations were performed with 5-fold increase in noise intensity by changing clock amplitude (i.e., w = 1.5, c = 0~2 in Eq 2). (B-D) Representative simulation results when the clock amplitude was decreased 0.2-fold (B), under standard conditions (C), and increased 2-fold (D). (E) Effect of noise on somite size. The formation of 200 somites was simulated by changing the clock amplitude from 0 to 2-fold. The data represent the means and standard deviations. (TIF) [file pcbi.1006579.s005.tif]

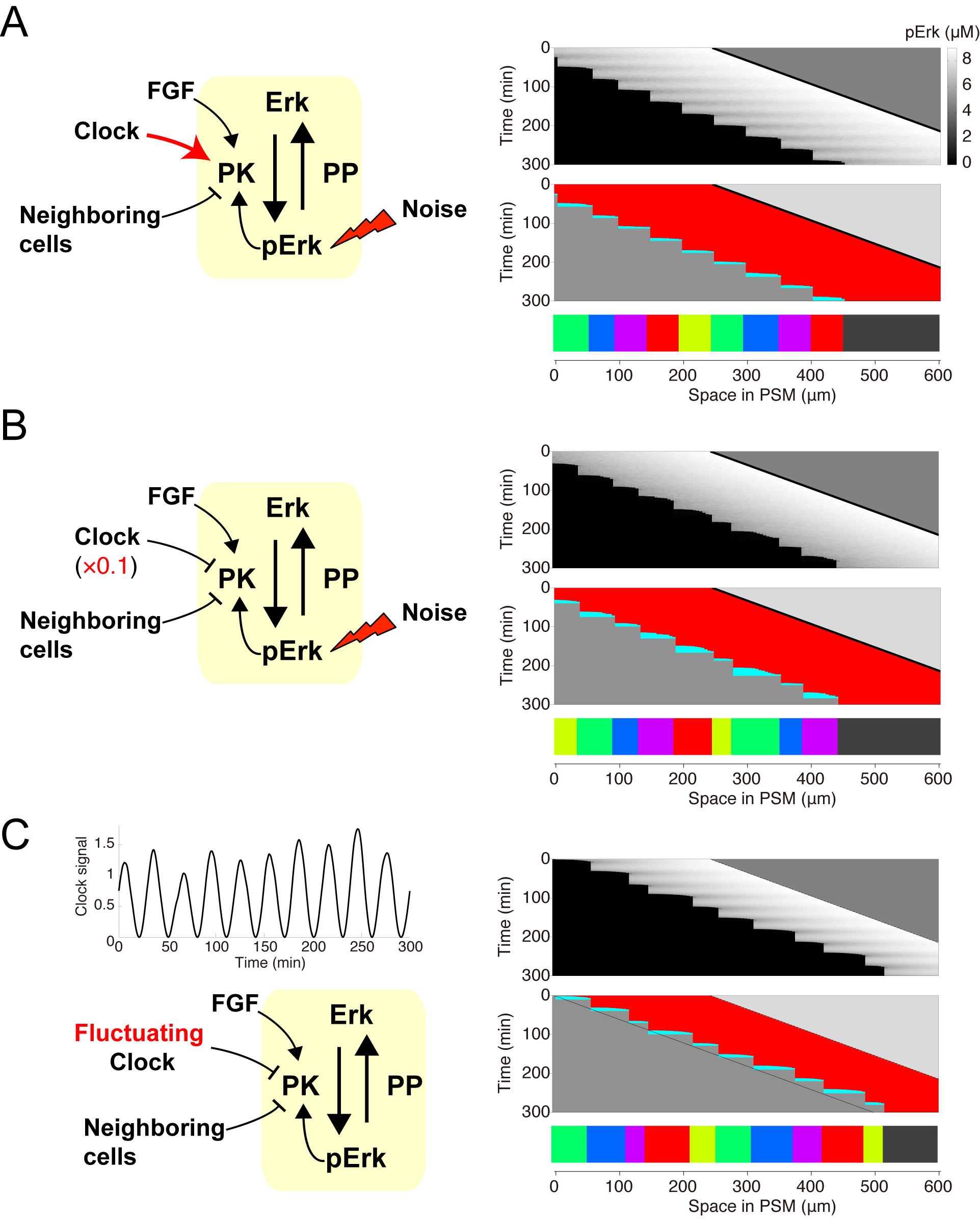

Supplement: S5 Fig — (A) Simulation result with a positive clock effect (i.e., c = −0.2 in Eq 2). Note that somites are normally formed, even when ERK is positively regulated by the clock. (B) Simulation result with a reduced clock amplitude (i.e., c = 0.02 in Eq 2). Note that somites are irregularly formed as seen in a clock-deficient embryo. (C) Simulation result with fluctuation of clock amplitude. Temporal change in clock amplitude was generated by Ornstein-Uhlenbeck process (inset). (TIF) [file pcbi.1006579.s006.tif]

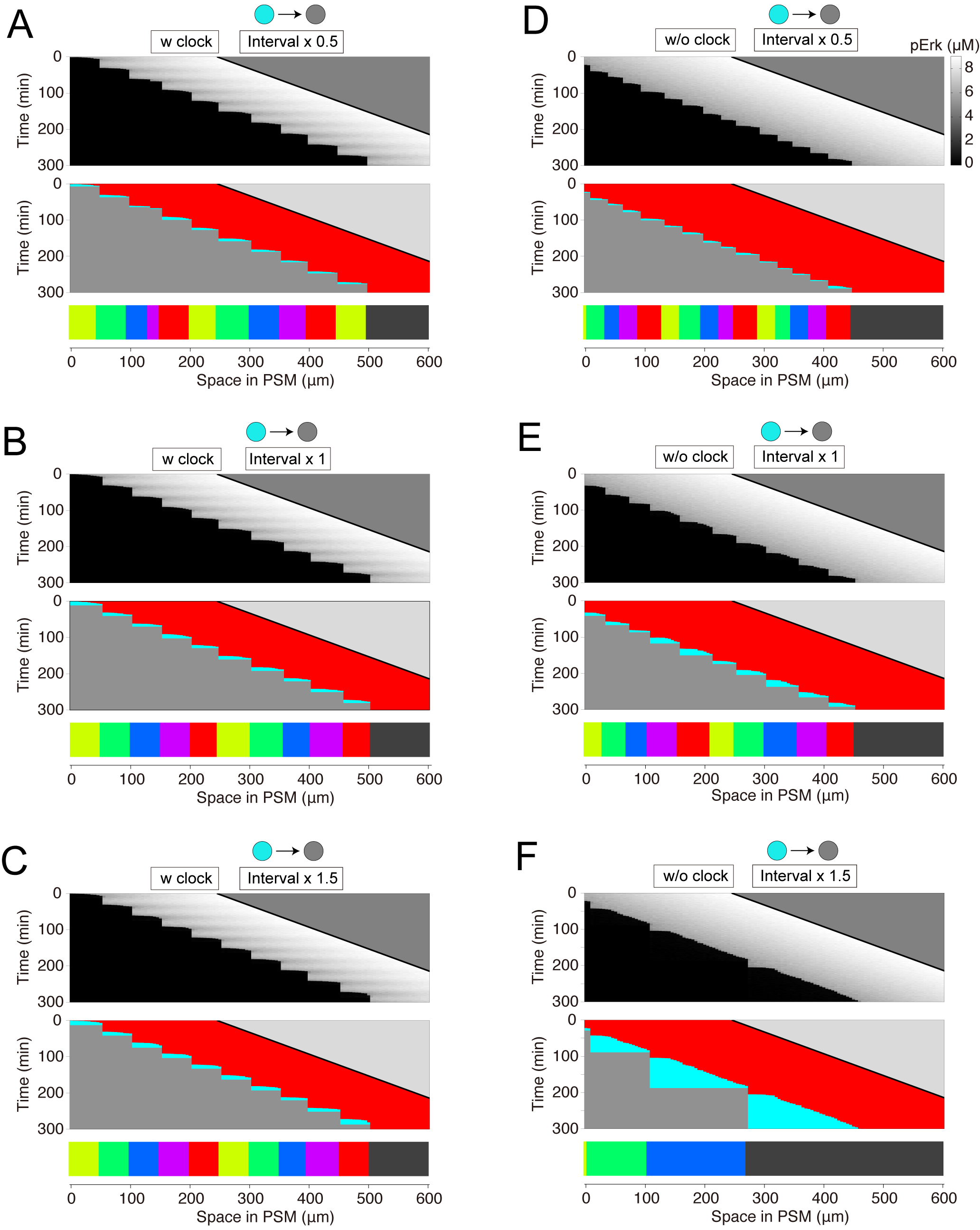

Supplement: S6 Fig — Simulations were performed with a 5-fold increase in noise intensity by changing the time interval for somite differentiation in both the presence and absence of the clock (i.e., w = 1.5, c = 0 or 1 in Eq 2). (A-C) Representative simulation results in the presence of the clock when the time interval decreased 0.5-fold (A), under standard conditions (B), and increased 2-fold (C). (D-F) Representative simulation results in the absence of the clock when the time interval decreased 0.5-fold (D), under standard conditions (E), and increased 2-fold (F). (TIF) [file pcbi.1006579.s007.tif]
